# Unruptured Intracranial Aneurysm Risk Scores Underperform in Predicting Subsequent Rupture: A Retrospective Single-Center Study

**DOI:** 10.3390/neurolint17110189

**Published:** 2025-11-20

**Authors:** Kamil Krystkiewicz, Aleksander Kowal, Magdalena Krystkiewicz-Orzechowska, Filip Arczewski, Karol Dziedzic, Marcin Tosik

**Affiliations:** 1 Department of Neurosurgery and Neurooncology, Copernicus Memorial Hospital, 93-513 Lodz, Poland; aleksanderwkowal@gmail.com (A.K.); farczewski@interia.pl (F.A.); karol.dziedzic@student.umed.lodz.pl (K.D.); marcin.tosik@wp.pl (M.T.); 2 Department of Molecular Carcinogenesis, Medical University of Lodz, 90-752 Lodz, Poland

**Keywords:** intracranial aneurysm, rupture risk, PHASES, ELAPSS, UIATS, subarachnoid hemorrhage

## Abstract

**Background/Objectives:** Risk-stratification tools, including the PHASES, UIATS, and ELAPSS, are commonly used to guide management of incidentally detected unruptured intracranial aneurysms (UIAs), but their predictive accuracy in real-world settings remains unclear. This study evaluated how these scores would have advised treatment in patients who subsequently presented with aneurysmal subarachnoid hemorrhage (aSAH). **Methods:** We retrospectively analyzed adults treated for aSAH at Copernicus Memorial Hospital (Łódź, Poland) between January 2022 and June 2024. For each ruptured aneurysm, we calculated PHASES (5-year rupture risk), UIATS recommendation, and ELAPSS (5-year growth risk) as if the lesion had been detected incidentally. Identical assessments were performed for UIAs that remained unruptured. Discrimination for rupture was evaluated using receiver-operating characteristic analysis (AUC). **Results:** Of 180 aneurysms (mean age 66.9 ± 11.3 years), 103 (57%) were ruptured. Patients with ruptured aneurysms were significantly older (69.9 vs. 64.0 years; *p* = 0.003), while sex, hypertension, smoking, and aneurysm morphology did not differ significantly. UIATS more frequently favored conservative management in ruptured aneurysms (56.3% vs. 39.0%; *p* = 0.046). PHASES (1.6% vs. 1.6%) and ELAPSS (3-year: 14.5% vs. 12.6%; 5-year: 22.6% vs. 20.0%) showed no significant differences between groups. Age was the only independent predictor of rupture (OR = 1.05/year; *p* < 0.001). The model’s cross-validated AUC was 0.731. **Conclusions:** Most ruptured aneurysms would not have been recommended for treatment based on UIATS. PHASES, ELAPSS, and UIATS did not reliably discriminate between ruptured and unruptured aneurysms, emphasizing the need for more precise and individualized risk assessment tools.

## 1. Introduction

Unruptured intracranial aneurysms (UIAs) occur in an estimated 3–5% of the general population and constitute a potentially life-threatening cerebrovascular condition [1,2]. Evidence from the ISUIA study indicates that most aneurysms remain asymptomatic, with rupture risk primarily influenced by location and size [3]. The widespread use of advanced neuroimaging has led to increased incidental detection of UIAs, prompting neurosurgeons to weigh the risks of prophylactic intervention against the serious consequences of subarachnoid hemorrhage [4,5]. Craniotomy with clipping is associated with higher neurological morbidity compared to endovascular approaches for both ruptured [6,7] and unruptured lesions [8]; however, endovascular therapy is linked to higher rates of re-rupture and retreatment. Therefore, optimal management requires an individualized assessment of hemorrhage risk versus procedural risk, considering patient age, comorbidities, aneurysm morphology, and anatomical location.

To support this complex decision-making process, several risk-stratification tools have been developed. The PHASES score, derived from six prospective cohorts, estimates the 5-year rupture risk using six easily accessible variables: population, hypertension, age, aneurysm size, prior aneurysmal hemorrhage, and location [5]. Although it has been externally validated [9], PHASES omits essential factors, such as smoking and multiplicity [10]. The Unruptured Intracranial Aneurysm Treatment Score (UIATS) was developed through a Delphi consensus of multidisciplinary experts; its 29 variables yield a recommendation for treatment, conservative management, or equivocal guidance [11]. External validation studies, however, have demonstrated inconsistencies in UITAS [12]. Recognizing that aneurysm growth is a strong indicator of rupture risk, Backes and colleagues proposed the ELAPSS model to predict the risk of 3- and 5-year enlargement risk [13].

Many cerebrovascular specialists believe that current risk scores often underestimate hemorrhage risk, especially when subarachnoid hemorrhage occurs in aneurysms that would not have been treated based on these scores. Several retrospective analyses support this view. However, because the natural history of cerebral aneurysms remains only partly understood and existing tools are imperfect, applying published guidelines to real-world practice is challenging.

The present study assesses PHASES, UIATS, and ELAPSS in a cohort of patients who ultimately suffered aneurysmal subarachnoid hemorrhage, simulating the treatment decisions that might have been made if these aneurysms had been found incidentally.

## 2. Materials and Methods

**Figure 1:**
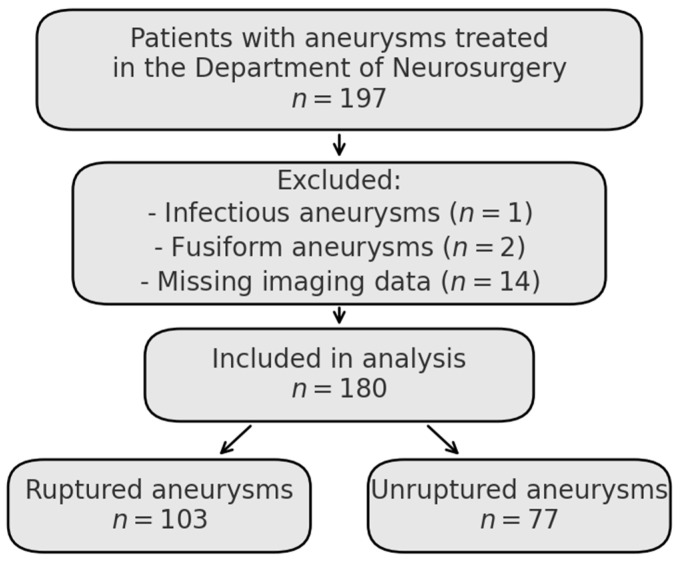
Flowchart of patient selection for the final study cohort. Consecutive aneurysms treated at the Department of Neurosurgery were initially considered. After applying exclusion criteria including infectious aneurysms, fusiform morphology, and missing imaging data, a total of 180 aneurysms were included in the final analysis. These were stratified by rupture status (ruptured vs. unruptured).

A retrospective analysis was conducted using the medical records of adult patients diagnosed and treated for aneurysmal subarachnoid hemorrhage in the Department of Neurosurgery and Neurooncology in Copernicus Memorial Hospital from January 2022 to June 2024. Consecutive patients with intracranial aneurysms who received treatment in this department were identified retrospectively. Exclusion criteria included infectious aneurysms, fusiform morphology, and incomplete imaging data. The final study cohort included 180 aneurysms, as shown in the flowchart (Figure 1). The database was reviewed to ensure fulfillment of all PHASES, UIATS, and ELAPSS score variables were available.

For the unruptured cohort, aneurysms were detected incidentally by outpatient non-emergency imaging (either computed tomography angiography or magnetic resonance angiography). Only aneurysms that were subsequently treated at the Department of Neurosurgery were considered. Details regarding the procedures performed were collected. Patients with incomplete data, lacking complete data for all scoring variables, were excluded.

Senior neurosurgeons (KK and MT), each with experience in treating complex neurovascular cases, evaluated recommendations and score results. In cases with multiple aneurysms, only one aneurysm per patient was included in the analysis—specifically, the ruptured aneurysm in patients with subarachnoid hemorrhage or the treated aneurysm in patients with unruptured lesions—to maintain the independence of observations and avoid clustering bias. All patients provided informed consent for the scientific use of their medical data. Ethical review and approval were waived for this study due to its retrospective design, use of fully anonymized clinical data, and the fact that, under regional Institutional Review Board policy, such analyses do not meet the definition of human-subjects research and therefore do not require formal IRB approval, in accordance with the exemption criteria outlined in 45 CFR 46.104.

All statistical calculations were performed with Python 3.10 (packages: pandas, SciPy (an open-source scientific computing library for Python, community-developed, supported by NumFOCUS, USA), statsmodels, and scikit-learn). Continuous variables were inspected for normality by the Shapiro-Wilk test. All metrics (age, largest dimension, size ratio, aspect ratio, maximum height, PHASES, and ELAPSS scores) deviated significantly from a Gaussian distribution (*p* < 0.05). Results were presented as the mean ± standard deviation (SD); the Mann-Whitney U test assessed between-group differences. Categorical variables (sex, smoking status, arterial hypertension, multiplicity, UIATS recommendation) are reported as counts and percentages. Binary variables were compared using the χ^2^ test without Yates’ continuity correction. The three-level UIATS variable was first analyzed with a global χ^2^ test, followed, when indicated, by 2 × 2 post hoc tests adjusted for multiple comparisons (Bonferroni).

A multivariable logistic regression model was developed to identify independent predictors of aneurysm rupture. Variables for the model were selected a priori based on clinical relevance and literature, and included age (continuous), sex (male vs. female), the largest aneurysm dimension in millimeters (continuous), arterial hypertension (yes/no), active smoking (yes/no), PHASES score (as continuous percentage risk), and morphological irregularity (regular vs. irregular). The ELAPSS score was excluded to avoid collinearity with the PHASES score. The model used complete case analysis, yielding a final sample size of 163 aneurysms. Odds ratios (ORs) with 95% confidence intervals (CIs) and associated *p*-values were calculated for each predictor.

Internal validation of the model’s discriminative ability was performed using bootstrap resampling with 1000 iterations. The area under the receiver operating characteristic (ROC) curve (AUC) was calculated, with its confidence interval. The optimal threshold for classifying aneurysms as ruptured or unruptured was determined by Youden’s index on the ROC curve, with sensitivity and specificity reported. The Hosmer-Lemeshow goodness-of-fit test was used to assess model calibration. The model was fitted with Statsmodels (Python 3.10) and scikit-learn (v1.3.0) for model evaluation and bootstrapping (using sklearn.utils.resample). Robustness was confirmed with an L1-Regularized version, which produced a similar estimate. Collinearity was negligible (all VIF < 2). Calibration was acceptable (Hosmer–Lemeshow χ^2^ = 4.5, df = 8, *p* = 0.81).

## 3. Results

In the final group (*n* = 180), there were 103 ruptured aneurysms (57%) and 77 unruptured lesions (43%). Patients with ruptured aneurysms were older (69.9 ± 10.3 vs. 64.0 ± 13.5 years; *p* = 0.003, Mann-Whitney U). Sex distribution was similar between groups (female/male, 71.8%/28.2% vs. 72.7%/27.3%; *p* = 1.000, χ^2^), as were the rates of current smoking (35.4% vs. 20.0%; *p* = 0.391) and arterial hypertension (70.3% vs. 67.2%; *p* = 0.309). The mean largest diameter (6.2 ± 3.6 mm vs. 5.9 ± 3.1 mm), size ratio (2.7 ± 1.5 vs. 2.8 ± 1.6), aspect ratio (1.1 ± 0.6 vs. 1.2 ± 0.6), and maximum height (5.0 ± 3.3 mm vs. 4.8 ± 2.6 mm) showed no significant differences (all *p* > 0.05, Table 1). Analysis of the PHASES scores revealed a median of 4.0 points for the entire cohort. When stratified by rupture status, the median score was 5.0 for the ruptured group and 4.0 for the unruptured group.

The distribution of aneurysm sizes varied with rupture status (Table 2). Among ruptured aneurysms, most (63/124; 50.9%) measured ≤ 5 mm in diameter, compared to 23/56 (41.2%) in the unruptured group. Very small aneurysms (≤3 mm) made up 18.6% of ruptured lesions, versus 14.3% of unruptured lesions. Aneurysms measuring 3–5 mm occurred in 32.3% of ruptured and 26.8% of unruptured cases. Larger aneurysms (6–15 mm) were more common in unruptured cases (42.9%) than in ruptured ones (30.7%). Aneurysms ≥ 15 mm represented a small portion overall but were slightly more frequent in ruptured cases (4.8%) compared to unruptured (1.8%).

The distribution of UIATS recommendations varied between ruptured and unruptured aneurysms (global χ^2^ = 55.9, df = 2, *p* = 0.042). To clarify which category was influencing this difference, three Bonferroni-adjusted 2 × 2 post hoc tests were performed. The recommendation “conservative management” was more frequent in the ruptured group (58/103, 56.3%) than in the unruptured group (30/77, 39.0%), but this difference did not reach statistical significance after correction (adjusted *p* = 0.046; Bonferroni-corrected threshold = 0.0167). In contrast, neither the “neurosurgeon’s decision” (30/103, 29.1% vs. 26/77, 33.8%; adjusted *p* = 1.000) nor “treatment” (9/103, 8.7% vs. 15/77, 19.5%; adjusted *p* = 0.116) showed a statistically significant difference after correction for multiple comparisons.

The comparison of rupture prediction scores revealed that neither PHASES nor ELAPSS was able to distinguish between ruptured and unruptured aneurysms. Specifically, the mean PHASES 5-year rupture probability was 1.6 ± 2.6% in ruptured lesions and 1.6 ± 2.3% in unruptured ones (*p* = 0.790, Mann-Whitney U), indicating nearly identical risk assessments across groups. Likewise, ELAPSS, which predicts aneurysm growth rather than rupture, produced overlapping estimates: the 3-year growth risk averaged 14.5 ± 10.2% for ruptured aneurysms versus 12.6 ± 8.0% for unruptured (*p* = 0.499), and the 5-year risk was 22.6 ± 14.3% versus 20.0 ± 11.6% (*p* = 0.507).

These findings suggest that, within this cohort, the current scoring systems, which combine clinical and radiological factors, failed to identify features ultimately linked to aneurysm rupture. Age was the only variable showing a statistically significant difference between the ruptured and unruptured groups.

**Figure 2:**
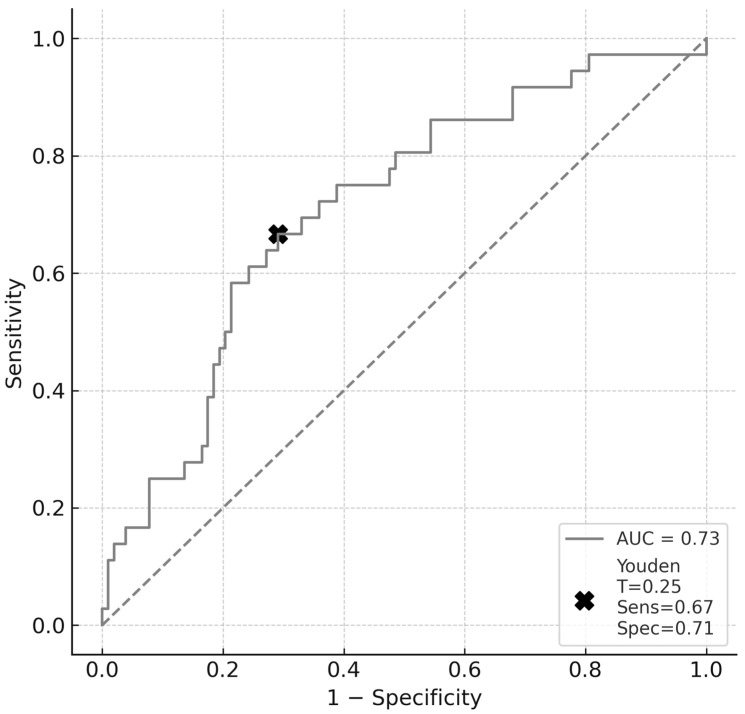
The gray ROC curve illustrates the ability of the multivariable model to discriminate between ruptured and unruptured aneurysms, as evaluated using bootstrap resampling with 1000 iterations (AUC = 0.731; 95% CI: 0.638–0.817). The X marker denotes Youden’s optimal threshold (cut-off = 0.25), at which the model achieves a sensitivity of 0.67 and a specificity of 0.71. AUC—area under the curve, T—threshold, Sens—sensitivity, Spec—specificity.

In the multivariable logistic regression model that controlled for age and included sex, the largest aneurysm dimension, arterial hypertension, smoking, PHASES rupture risk score, and morphological irregularity, only age retained an independent, significant association with aneurysm rupture (adjusted OR = 1.05 per year; 95% CI: 1.02–1.08; *p* < 0.001) (Table 3). The ELAPSS score was excluded from the model due to collinearity with the PHASES score. None of the other covariates reached statistical significance following adjustment: sex (OR = 1.20; 95% CI: 0.55–2.65; *p* = 0.65), largest aneurysm dimension (OR = 1.02 per mm; 95% CI: 0.93–1.12; *p* = 0.64), hypertension (OR = 0.88; 95% CI: 0.38–2.02; *p* = 0.76), smoking (OR = 1.34; 95% CI:0.49–3.63); *p* = 0.566), PHASES score (OR = 0.94 per percentage point; 95% CI: 0.78–1.12; *p* = 0.48), and irregular shape (OR = 0.82; 95% CI: 0.44–1.55; *p* = 0.54). The model was internally validated using bootstrap resampling with 1000 iterations, yielding an AUC of 0.731 (95% CI: 0.638–0.817), which is consistent with moderate discriminative performance (Figure 2). Using Youden’s index, we identified the optimal probability threshold as 0.25. At this cut-off, the model demonstrated a sensitivity of 0.67 and specificity of 0.71, resulting in a Youden index of 0.38, which reflects a fair discriminative capacity. Model calibration remained satisfactory, as indicated by a Hosmer-Lemeshow *p*-value of 0.64.

Analysis of the UIATS recommendations revealed significant differences in management suggestions between ruptured and unruptured aneurysms. Among unruptured aneurysms, conservative treatment was recommended in 56 cases (73.7%), a neurosurgical decision was made in 15 cases (19.7%), and surgical treatment was considered in 14 cases (18.4%). In contrast, among ruptured aneurysms, conservative management was suggested in 55 cases (53.4%), a neurosurgical decision was made in 40 cases (38.8%), and surgical treatment was recommended in only 8 cases (7.8%). Chi-square tests performed separately for each recommendation category revealed a statistically significant difference only for the “neurosurgeon decision” category (*p* = 0.002), which was more frequent among patients with ruptured aneurysms; no significant differences were observed in the “conservative” (*p* = 0.628) or “treatment” (*p* = 0.329) categories.

**Figure 3:**
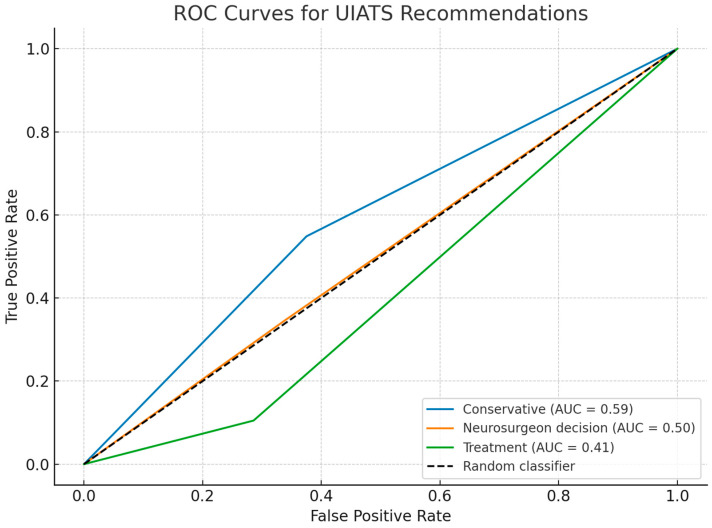
Receiver Operating Characteristic (ROC) curves assessing the ability of UIATS recommendation categories—“conservative”, “neurosurgeon decision”, and “treatment”—to discriminate between ruptured and unruptured aneurysms. UIATS—unruptured intracranial aneurysm treatment score.

Overall, 92% (95 out of 103) of ruptured aneurysms were not explicitly classified for treatment by the UIATS algorithm but fell into either the “neurosurgeon decision” or “conservative management” categories (Table 4). Although primarily designed to guide management decisions rather than predict aneurysm rupture, the UIATS incorporates multiple variables known to be associated with an increased risk of intracranial bleeding. Therefore, ROC analysis was performed to evaluate the ability of UIATS recommendation categories to discriminate between ruptured and unruptured aneurysms. The results revealed limited predictive value across all categories; the “conservative” category demonstrated the highest discriminatory ability (AUC = 0.59), followed by the “neurosurgeon decision” category (AUC = 0.50) and the “treatment” category (AUC = 0.41). However, none of the categories achieved acceptable AUC levels (>0.7), indicating that UIATS recommendations alone have limited ability to predict rupture status in this cohort (Figure 3).

The comparative analysis of PHASES and ELAPSS scores between ruptured and unruptured aneurysms is summarized in Table 1. No statistically significant differences were found in PHASES rupture risk estimates between the two groups (1.6 ± 2.6% vs. 1.6 ± 2.3%, *p* = 0.790). Similarly, ELAPSS growth risk scores did not differ significantly between the two groups. The mean 3-year ELAPSS risk was 14.5 ± 10.2% in ruptured versus 12.6 ± 8.0% in unruptured aneurysms (*p* = 0.499), while the 5-year ELAPSS risk was 22.6 ± 14.3% and 20.0 ± 11.6%, respectively (*p* = 0.507). These results indicate that neither PHASES nor ELAPSS was able to discriminate between ruptured and unruptured aneurysms in this cohort.

**Figure 4:**
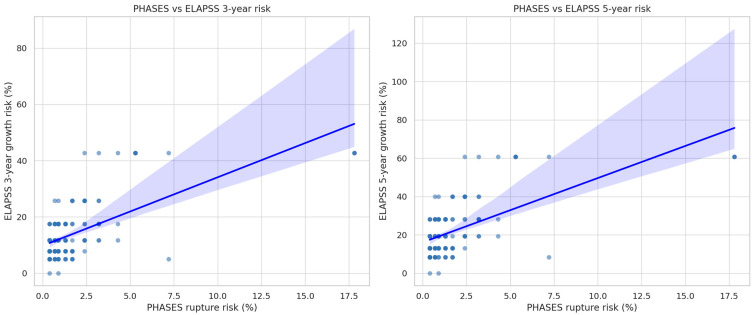
Scatterplots illustrating the correlation between PHASES score (aneurysm rupture risk) and ELAPSS score (aneurysm growth risk) at 3 years (**left panel**) and 5 years (**right panel**). The regression line indicates a weak but statistically significant positive association between the rupture and growth risk scores (Spearman’s ρ = 0.21 for 3-year ELAPSS and ρ = 0.28 for 5-year ELAPSS; both *p* < 0.05).

Spearman’s rank-order correlation was performed to assess the relationship between PHASES score and both the 3-year and 5-year ELAPSS growth risk scores (Figure 4). The analysis revealed a moderate positive correlation between PHASES and the 5-year ELAPSS risk (ρ = 0.488, *p* < 0.0001), as well as a weaker but still statistically significant correlation between PHASES and the 3-year ELAPSS risk (ρ = 0.420, *p* < 0.0001).

**Figure 5:**
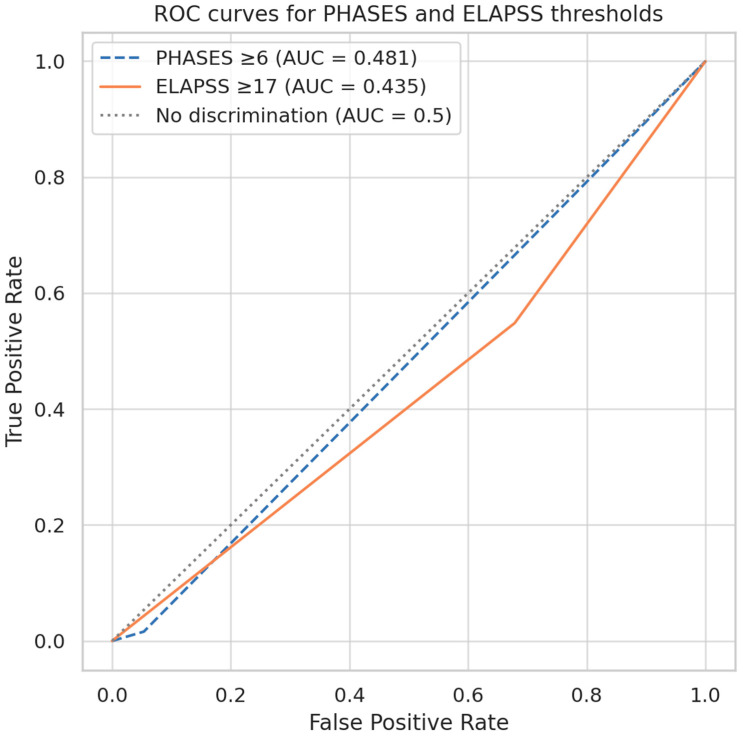
ROC curves for PHASES (cutpoint ≥ 6) and ELAPSS 5-year risk (cutpoint ≥ 17) in predicting aneurysm rupture. Both showed low discriminative power (AUC = 0.481 and 0.435, respectively).

Further evaluation of predefined cutoff points for PHASES and ELAPSS scores to differentiate ruptured from unruptured aneurysms revealed limited predictive performance. For the PHASES score, a cut-off value of ≥6 points (corresponding to an estimated 5-year rupture risk of approximately 1.7%) resulted in an AUC of 0.481 (Figure 4). This threshold demonstrated a sensitivity of 21.6%, a specificity of 84.1%, a positive predictive value (PPV) of 37.0%, and a false-negative rate of 78.4% (Table 5). Similarly, ELAPSS score, evaluated at a cut-off of ≥17 points (corresponding to a 5-year growth risk of approximately 28.1%), yielded an AUC of 0.435 (Figure 5). This threshold demonstrated a sensitivity of 31.4%, specificity of 82.5%, PPV of 44.4%, and a false-negative rate of 68.6% (Table 5).

## 4. Discussion

The management of UIAs remains a subject of clinical debate. Each therapeutic intervention carries a risk of complications; therefore, treatment decisions require careful consideration and evaluation. This process is complicated by a substantial body of often conflicting data on risk factors and management guidelines, which can impede definitive decision-making in clinical practice.

Despite advances in understanding the natural history of cerebral aneurysms, informed by large-scale trials such as ISUIA [3] and predictive scores like PHASES, ELAPSS, and UIATS, ultimate therapeutic decisions largely depend on the clinician’s experience. Our study demonstrates that these standard clinical and morphological models, including PHASES, ELAPSS, and UIATS, have limited accuracy in distinguishing between ruptured and unruptured aneurysms in real-world settings. Although widely referenced, they lack sufficient classification accuracy, raising concerns about their standalone reliability. The PHASES score, despite being validated in large prospective datasets, yielded nearly identical rupture risk estimates for ruptured and unruptured aneurysms in our cohort, questioning its usefulness in urgent decision-making, especially for patients outside the narrowly defined high-risk groups (e.g., Finnish or Japanese populations). The ELAPSS score, designed to predict aneurysm growth rather than rupture, demonstrated poor discriminatory power and only moderate correlation with PHASES, highlighting the differing assessment domains of these two scores. The UIATS algorithm, designed to guide management decisions, often classifies ruptured aneurysms into “conservative” or “neurosurgeon decision” groups. Most ruptured aneurysms do not clearly meet treatment criteria, indicating a disconnect between recommendations and actual rupture risk.

Traditional size-based guidelines, including ISUIA’s recommendation for conservative management of aneurysms smaller than 7 mm, are challenged by clinical practice, where small, ruptured aneurysms are frequently treated. This size criterion is also a fundamental component of risk scores. However, this paradigm is challenged by clinical practice, in which neurosurgeons treat patients with SAH caused by the rupture of small aneurysms—those that would not have qualified for prophylactic treatment based on the aforementioned guidelines. Our data and other studies confirm that many ruptured aneurysms are small (<6 mm), a size group often approached conservatively per guidelines. In ISAT, 51% of endovascular procedures and 53% of surgical clippings were performed on aneurysms measuring ≤5 mm [14]. Similarly, in the BRAT trial, the median size of ruptured aneurysms was 6.0 mm in both the surgical and endovascular treatment groups [15]. In our study, the median size of a ruptured aneurysm was similar, estimated at 6.2 ± 3.6 mm. Moreover, nearly 51% of the ruptured cases were smaller than 6 mm, which, according to the ISUIA recommendation, would be approached conservatively. Our findings align with an extensive retrospective analysis of 628 SAH cases by Rutledge and colleagues, who found that the median size of a ruptured aneurysm was 5.3 mm, and 48% of them were smaller than 5 mm [16]. Additionally, their study reported a median PHASES score of 5, indicating a 1.3% 5-year risk of rupture. The median 5-year risk in our analysis was similar, at 1.6%, matching the results of the aforementioned publication. 

Despite the predominance of small aneurysms (<6 mm) among ruptured cases in our cohort, traditional models, such as PHASES, fail to capture the rupture risk in these lesions adequately. This paradox may be partly explained by biomechanical and hemodynamic factors that transcend size-based classification. Several studies indicate that abnormal wall shear stress, especially when combined with high flow instability or low oscillatory shear index, can lead to focal wall degeneration, even in small aneurysms [17,18]. Moreover, complex morphologies, such as lobulations or daughter sacs, can create flow impingement zones that intensify local stress. In such conditions, thin-walled regions, which may not be readily apparent in macroscopic morphological assessments, can become vulnerable to rupture [19,20]. Our observations, combined with the limited discriminatory power of models based solely on clinical and morphological variables, underscore the need for next-generation predictive tools. These tools should incorporate hemodynamic and wall integrity assessments, potentially through computational fluid dynamics or advanced imaging techniques such as high-resolution vessel wall MRI. Such advancements are crucial for enhancing the accuracy of risk stratification, particularly for small aneurysms that would otherwise be classified as low risk by conventional criteria.

The apparent discrepancy between our findings regarding the size of ruptured aneurysms and ISUIA classical recommendations can be interpreted as evidence of shifting population dynamics over the last few decades. A compelling hypothesis is that successful public health interventions have altered the natural history of intracranial aneurysms. For instance, Korja et al. provided strong evidence for this trend in their analysis of consecutive SAH cases between 1989 and 2009 [20]. They observed that the median size of ruptured aneurysms significantly decreased over these 20 years, with a notable 15% reduction in patients younger than 50 years old [21]. Hence, while ISUIA guidelines were groundbreaking, they may need to be recalibrated to reflect the characteristics of the contemporary patient population, where smaller aneurysms appear to pose a greater relative threat than previously estimated. This is more crucial, given that known and used scoring systems (PHASES, ELAPSS, UIATS) incorporate the size of the aneurysms in the results algorithm.

Our study revealed that the estimated 5-year risk of rupture via PHASES in both ruptured and unruptured aneurysms suggests potential underestimation of bleeding risk in certain clinical subgroups. A number of aneurysms ruptured despite having low predicted risk values, indicating that the variables included in the score may not fully capture rupture-prone lesions in a focused clinical population. This limitation has been noted in previous literature, where several authors criticized PHASES for omitting important clinical or morphological features such as smoking status or aneurysm enlargement [22,23]. Additionally, the scoring system assigns considerable weight to geographic population categories (e.g., Japanese, Finnish), which may improve calibration in global datasets but reduces accuracy when applied to more uniform cohorts.

Unlike the study by Bijlenga et al. [24], which reported significantly higher PHASES scores in ruptured aneurysms (mean 4.73 vs. 2.92), we found no statistically significant difference between groups. This discrepancy may reflect differences in cohort composition and methodology: Bijlenga’s work was based on a large, population-based cross-sectional study, whereas our dataset represents a single-center, neurosurgical cohort. Notably, even in their study, ruptured aneurysms frequently exhibited low PHASES scores, supporting our observation that commonly used PHASES thresholds (e.g., ≥6 points) poorly identify rupture retrospectively. These converging findings underscore the need for refinement or supplementation of current scoring systems when applied to clinical decision-making in real-world settings.

Based on our results, we attempted to evaluate a cut point for bleeding risk, which could categorize patients into two distinct groups: those with a high risk of hemorrhage and those with a low risk. However, the low true predictive potential, with a high proportion of false negatives (above 50% of cases), revealed that neither score was sufficiently precise to identify the risk of rupture reliably. What should be noted is that the UIAs group is biased by the fact that the physician electively chooses patients for aneurysm repair. Small aneurysms, especially those < 5 mm, were preferably approached more conservatively by serial radiological examinations than scheduled for treatment. This fact should be considered when discussing the presented results, as the criterion of size is one of the components of both scoring systems. Still, the size of the ruptured aneurysms remains unbiased and significantly lower than the ISUIA threshold.

This study highlights the limited ability of the UIATS algorithm to identify aneurysms at risk of rupture. Despite including many known risk factors, UIATS retrospectively classified 92% of ruptured aneurysms as either “conservative management” or “neurosurgeon decision,” with only 8% clearly indicated for surgical treatment. This discrepancy suggests that UIATS may underestimate the urgency in high-risk cases. Although not intended to predict rupture, UIATS overlaps with rupture risk variables, and its poor discriminatory performance raises concern regarding its reliability in guiding management. The overuse of the “neurosurgeon decision” category in ruptured cases may reflect algorithm’s uncertainty where clinical intuition proves more accurate. Moreover, when UIATS was evaluated as a potential predictor of aneurysm rupture, its performance proved suboptimal, showing a substantial mismatch in our cohort. ROC analysis demonstrated poor prognostic accuracy across all recommendation categories. Although the “neurosurgeon decision” group exhibited the highest sensitivity for predicting subarachnoid hemorrhage, it remains the most ambiguous category, relying heavily on individual clinical judgment rather than standardized criteria. Importantly, UIATS was initially designed to support treatment decision-making in unruptured aneurysms, not to predict rupture risk. Its limited discriminatory ability in this context reinforces the notion that it is not suited for prognostic purposes.

The results of our multivariable logistic regression analysis warrant cautious interpretation. Although age emerged as the only independent predictor of aneurysm rupture, the model’s explanatory power remains limited. Each additional year of life increased the odds of rupture by 5%, yet this alone does not justify a strong clinical inference, particularly given the complexity of aneurysm pathophysiology. The fact that traditional factors, such as aneurysm size, morphology, hypertension, smoking, and even the PHASES score, did not retain statistical significance after adjustment may be partly explained by the limited sample size, the retrospective design, and potential collinearity between predictors. These findings may reflect model instability rather than a definitive absence of effect.

While the model achieved moderate discrimination (AUC = 0.731; 95% CI: 0.638–0.817), caution is warranted since such values can occur with weak classifiers, especially in imbalanced datasets. More importantly, the model’s utility as a clinical tool is debatable. Although calibration was acceptable (Hosmer-Lemeshow χ^2^ = 4.5, df = 8, *p* = 0.81), discrimination alone is insufficient for decision-making in high-stakes scenarios, such as the management of aneurysms. At the optimal threshold (Youden index = 0.25), sensitivity and specificity reached 67% and 71%, respectively. However, these values imply a non-negligible risk of both false positives and false negatives, which may limit the clinical applicability of the model in its current form.

It is also worth noting that the PHASES score, despite being widely cited, performed poorly here. Its inclusion in the multivariable model did not improve predictive value, and it failed to differentiate between ruptured and unruptured aneurysms at a group level. This aligns with other reports highlighting the limitations of PHASES when retrospectively applied to ruptured cohorts. Although we deliberately excluded the ELAPSS score from the model to avoid collinearity, we also observed a weak correlation between PHASES and ELAPSS scores, raising further questions about the robustness and external validity of both tools.

In summary, while our analysis contributes to the ongoing debate surrounding risk stratification in intracranial aneurysms, it also highlights the persistent shortcomings of morphology- and sole clinical-based models. Future work should incorporate hemodynamic modeling, vessel wall imaging, or molecular biomarkers to improve predictive performance. Until such multimodal approaches are validated, existing scores should be used with caution, and individual patient factors should remain central to decision-making.

Our study has several limitations that warrant consideration. First, it was conducted at a single center in Central Europe (Poland), which may introduce selection bias in the treatment decision-making process. However, it is essential to emphasize that our analysis focused on a cohort of ruptured aneurysms, which are not subject to elective treatment selection. Nevertheless, validation of our findings in an external cohort is required to enhance their generalizability. While we performed internal validation using bootstrap resampling to mitigate optimism bias, the absence of external validation remains a significant weakness.

Differences in patient selection, imaging practices, and treatment thresholds across institutions may limit the generalizability of our results. Furthermore, population-specific factors matter: the PHASES score accounts for Finnish patients known to have a higher aneurysm rupture risk but treats other European populations as homogeneous. The Polish population likely lacks distinct genetic predispositions, with epidemiological data showing aneurysmal subarachnoid hemorrhage incidence aligned with global averages, excluding high-risk groups. Therefore, caution should be exercised when extrapolating our findings across varying demographic, genetic, or healthcare settings. Future multicenter studies involving more genetically diverse populations are necessary to confirm the broader relevance of these findings. However, our findings underscore not only the known limitations of PHASES, ELAPSS, and UIATS but also their inconsistent performance within the same clinical cohort, highlighting the urgent need for improved predictive strategies. Although we did not introduce a new scoring system, our comparative approach and real-world data provide valuable groundwork for future model refinement.

In conclusion, our findings underscore the extraordinary complexity of the aneurysm rupture process and highlight the shortcomings of current predictive models. None of the evaluated tools—PHASES, ELAPSS, or UIATS—demonstrated sufficient discriminatory ability in our cohort. Despite its widespread clinical use, the PHASES score failed to distinguish ruptured from unruptured aneurysms, with overlapping median values and poor classification metrics. The ELAPSS score, designed to predict aneurysm growth, showed no correlation with rupture status and was only weakly associated with PHASES. The UIATS system, although intended to guide treatment rather than predict rupture, frequently categorized ruptured aneurysms as “conservative” or left the decision to the treating physician, further underscoring its limitations in predicting rupture. These observations suggest that while PHASES and UIATS remain helpful frameworks in routine practice, their predictive capacity should not be overestimated. Clinicians should exercise caution when relying solely on such scores, especially in cases with borderline results. Each patient requires a thorough individual evaluation that integrates their clinical presentation, radiological characteristics, and, if applicable, emerging biomarkers or hemodynamic data. Future efforts should focus on refining or developing tools that more accurately reflect the multifactorial nature of aneurysm rupture risk.

There is thus an urgent need to investigate novel biomarkers that could enrich our risk assessment strategies. These include hemodynamic factors, such as wall shear stress [23], markers of an active inflammatory process in the aneurysm wall [24], and the patient’s genetic predispositions [25]. Incorporating these elements into future predictive models may be crucial to making them more precise tools for daily clinical practice. Moreover, subgroup analyses—particularly in very old patients (≥85 years), where aneurysm behavior and comorbidity profiles may differ significantly—represent a promising direction for future research. Such prospective, multicenter studies could provide a more nuanced understanding of rupture mechanisms and improve individualized management.

## 5. Conclusions

In this study, we showed that many ruptured intracranial aneurysms were small, challenging traditional size-based management guidelines. Established scoring systems, including PHASES, ELAPSS, and UIATS, had limited ability to identify high-risk aneurysms in the past, often underestimating the danger in cases that eventually resulted in hemorrhage. Our multivariable analysis found patient age to be the only independent predictor of rupture, surpassing the predictive value of other common factors like aneurysm size. However, a predictive model based on these standard variables had very low ability to distinguish high-risk cases, making it unreliable for clinical decisions. Overall, our findings highlight the inadequacy of current risk assessment tools and stress the urgent need to explore and incorporate new biomarkers, including hemodynamics, vessel wall inflammation, and genetic factors, to develop more accurate predictive models.

## Figures and Tables

**Table 1:** General demographics in the analyzed cohort. UIATS—unruptured intracranial aneurysm treatment score.

Variable	Ruptured (*n* = 103)	Unruptured (*n* = 77)	*p*-Value	Statistical Test
Age (years)	69.9 ± 10.3	64.0 ± 13.5	**0.003**	Mann–Whitney U
Largest dimension (mm)	6.2 ± 3.6	5.9 ± 3.1	0.931	Mann–Whitney U
Size ratio	2.7 ± 1.5	2.8 ± 1.6	0.844	Mann–Whitney U
Aspect ratio	1.1 ± 0.6	1.2 ± 0.6	0.341	Mann–Whitney U
Maximum height (mm)	5.0 ± 3.3	4.8 ± 2.6	0.815	Mann–Whitney U
PHASES rupture risk (%)	1.6 ± 2.6	1.6 ± 2.3	0.790	Mann–Whitney U
ELAPSS 3-yr growth risk (%)	14.5 ± 10.2	12.6 ± 8.0	0.499	Mann–Whitney U
ELAPSS 5-yr growth risk (%)	22.6 ± 14.3	20.0 ± 11.6	0.507	Mann–Whitney U
Sex Female Male	74 (71.8%) 29 (28.2%)	56 (72.7%) 21 (27.3%)	1.000	Chi-square
Smoking Yes No	17 (35.4%) 31 (64.6%)	6 (20.0%) 24 (80.0%)	0.391	Chi-square
Hypertension Yes No	52 (70.3%) 22 (29.7%)	43 (67.2%) 21 (32.8%)	0.309	Chi-square
UIATS—Conservative	58 (56.3%)	30 (39.0%)	0.046	χ^2^ test, 2 × 2, Bonferonni
UIATS—Neurosurgeon decision	30 (29.1%)	26 (33.8%)	1.000	χ^2^ test, 2 × 2, Bonferonni
UIATS—Treatment	9 (8.7%)	15 (19.5%)	0.116	χ^2^ test, 2 × 2, Bonferonni

**Table 2:** Aneurysm size distribution based on rupture status.

Aneurysm Diameter (mm)	Unruptured	Ruptured
**≤3**	8 (14.3%)	23 (18.6%)
**3–5**	15 (26.8%)	40 (32.3%)
**5–6**	8 (14.3%)	17 (13.7%)
**6–9**	17 (30.4%)	25 (20.2%)
**9–15**	7 (12.5%)	13 (10.5%)
**15–25**	1 (1.8%)	6 (4.8%)

**Table 3:** Association between clinical and morphological predictors and aneurysm rupture status from the final multivariable logistic regression model. OR—odds ratio. CI—confidence interval. Values are the mean ± SD or number (%). Continuous variables compared using the Mann-Whitney U test. Categorical variables compared using χ^2^ test (Fisher’s exact test where appropriate). For UIATS categories, the Bonferroni correction was applied.

Predictor	Adjusted OR (95% CI)	*p*-Value
Age (per year)	1.05 (1.02–1.08)	<0.001
Sex (male vs. female)	1.20 (0.55–2.65)	0.654
Largest dimension (mm)	1.02 (0.93–1.12)	0.633
Hypertension (yes vs. no)	0.88 (0.38–2.02)	0.768
Smoking (yes vs. no)	1.34 (0.49–3.63)	0.566
PHASES score (%)	0.94 (0.78–1.12)	0.463
Irregular shape (yes vs. no)	0.82 (0.44–1.55)	0.543

**Table 4:** Distribution of UIATS recommendations stratified by aneurysm rupture status. UIATS—unruptured intracranial aneurysm treatment score.

UIATS Recommendation	Unruptured	Ruptured	*p* Value (Chi^2^ Test)
Conservative	56 (73.7%)	55 (53.4%)	0.628
Neurosurgeon decision	15 (19.7%)	40 (38.8%)	0.002
Treatment	14 (18.4%)	8 (7.8%)	0.329

**Table 5:** An ROC evaluation of the predictive potential of both systems follows the summary of the cutpoint optimization for PHASES and ELAPSS scores. PPV—positive predictive value, NPV—negative predictive value.

Scale	Cut-Off	AUC	Sensitivity	Specificity	PPV	NPV	False Negatives	False Negatives (%)
PHASES	≥6	0.481	0.22	0.84	0.37	0.78	124	98.4%
ELAPSS	≥17	0.435	0.32	0.83	0.44	0.65	47	68.6%

## Data Availability

Data underlying the results presented in this article are not publicly available due to institutional regulations, but can be obtained from the corresponding author on request.

## Abbreviations

The following abbreviations are used in this manuscript:

UIA	Unruptured intracranial aneurysm
aSAH	Aneurysmal subarachnoid hemorrhage
PHASES	Population, Hypertension, Age, Size of aneurysm, Earlier SAH, Site of aneurysm (risk score)
UIATS	Unruptured Intracranial Aneurysm Treatment Score
ELAPSS	Earlier SAH, Location, Age, Population, Size, Shape (growth risk score)
ROC	Receiver operating characteristic
AUC	Area under the curve
OR	Odds ratio
CI	Confidence interval
SD	Standard deviation
χ^2^	Chi-square test
VIF	Variance inflation factor
